# Procarbazine, CCNU and vincristine (PCV) versus temozolomide chemotherapy for patients with low-grade glioma: a systematic review

**DOI:** 10.18632/oncotarget.25890

**Published:** 2018-09-14

**Authors:** Karim Hafazalla, Arjun Sahgal, Blessing Jaja, James R. Perry, Sunit Das

**Affiliations:** ^1^ Sidney Kimmel Medical College at Thomas Jefferson University, Philadelphia, PA, USA; ^2^ Li Ka Shing Knowledge Institute, St. Michael's Hospital, University of Toronto, Toronto, ON, Canada; ^3^ Department of Radiation Oncology, Sunnybrook Odette Cancer Centre, University of Toronto, Toronto, ON, Canada; ^4^ Division of Neurology, Sunnybrook Health Sciences Centre, University of Toronto, Toronto, ON, Canada; ^5^ Division of Neurosurgery, University of Toronto, Toronto, ON, Canada

**Keywords:** glioma, IDH, low-grade glioma, PCV, temozolomide

## Abstract

Low-grade gliomas (LGG) encompass a heterogeneous group of tumors that are clinically, histologically and molecularly diverse. Treatment decisions for patients with LGG are directed toward improving upon the natural history while limiting treatment-associated toxiceffects. Recent evidence has documented a utility for adjuvant chemotherapy with procarbazine, CCNU (lomustine), and vincristine (PCV) or temozolomide (TMZ). We sought to determine the comparative utility of PCV and TMZ for patients with LGG, particularly in context of molecular subtype. A literature search of PubMed was conducted to identify studies reporting patient response to PCV, TMZ, or a combination of chemotherapy and radiation therapy (RT). Eligibility criteria included patients 16 years of age and older, notation of LGG subtype, and report of progression-free survival (PFS), overall survival (OS), and treatment course. Level I, II, and III data were included. Adjuvant therapy with PCV resulted in prolonged PFS and OS in patients with newly diagnosed high-risk LGG. This benefit was accrued most significantly by patients with tumors harboring 1p/19q codeletion and IDH1 mutation. Adjuvant therapy with temozolomide was associated with lower toxicity than therapy with PCV. In patients with LGG with an unfavorable natural history, such as with intact 1p/19q and wild-type IDH1, RT/TMZ plus adjuvant TMZ may be the best option. Patients with biologically favorable high-risk LGG are likely to derive the most benefit from RT and adjuvant PCV.

## INTRODUCTION

Low-grade gliomas (LGG) comprise a small portion of all primary brain tumors, roughly 5 to 10%. These tumors typically arise in younger adults, between 25 and 45 years of age, whereas anaplastic and high-grade tumors are more common in older patients. The natural history of most cases of low-grade gliomas is relatively favorable; examination of untreated patients followed by serial MRI revealed an annual growth rate of 4 to 6 mm per year [1]. Previous studies had delineated features of increased risk and poorer natural history, including age over 40 years, astrocytic histology, tumor diameter 6cm or longer, tumor crossing the midline, and neurological deficit prior to surgery. These features define a group that we will refer to as high-risk LGG [2].

Previous convention differentiated diffuse grade II and III gliomas into two basic subtypes: oligodendroglioma and astrocytoma. A third hybrid category, oligoastrocytoma, was used for tumors showcasing both oligodendroglioma and astrocytoma characteristics. Over the last five years, our understanding of LGG has evolved significantly, with a shift from classification of these tumors based on histology towards stratification of risk based on molecular subtype [3, 4]. According to the 2016 WHO classification system, grade II oligodendroglioma is now defined by an isocitrate dehydrogenase (IDH) mutation with whole-arm codeletion of chromosomal arms 1p and 19q, whereas IDH mutation combined with intact 1p and 19q chromosomal status is classified as IDH-mutant astrocytoma. Histological LGG in the setting of wild-type IDH is distinguished from IDH-mutant LGG and described as harboring a particularly poor natural history. While histological grade continues to be a factor in treatment consideration, the prognoses of patients with these tumors has been shown to correlate more closely with molecular alterations than with grade [3, 5, 6].

Treatment of patients with high-risk LGG is directed toward improving on the natural history of the disease, that is, extending time to malignant transformation and overall survival, while limiting treatment-associated morbidities and neurologic disability. Recent evidence has documented a utility for adjuvant chemotherapy with procarbazine, CCNU (lomustine), and vincristine (PCV) or temozolomide (TMZ) in the management of LGG. Unfortunately, most of these studies predate contemporary stratification systems for LGG, and conclusions of published series of glioma need to be re-examined in the light of the shifts that are brought about by the new WHO classification. We sought to determine the comparative utility of PCV and TMZ for patients with LGG, particularly in the context of molecular subtype. This review summarizes published data on adjuvant chemotherapy with PCV or TMZ for patients with LGG.

## RESULTS

**Figure 1:**
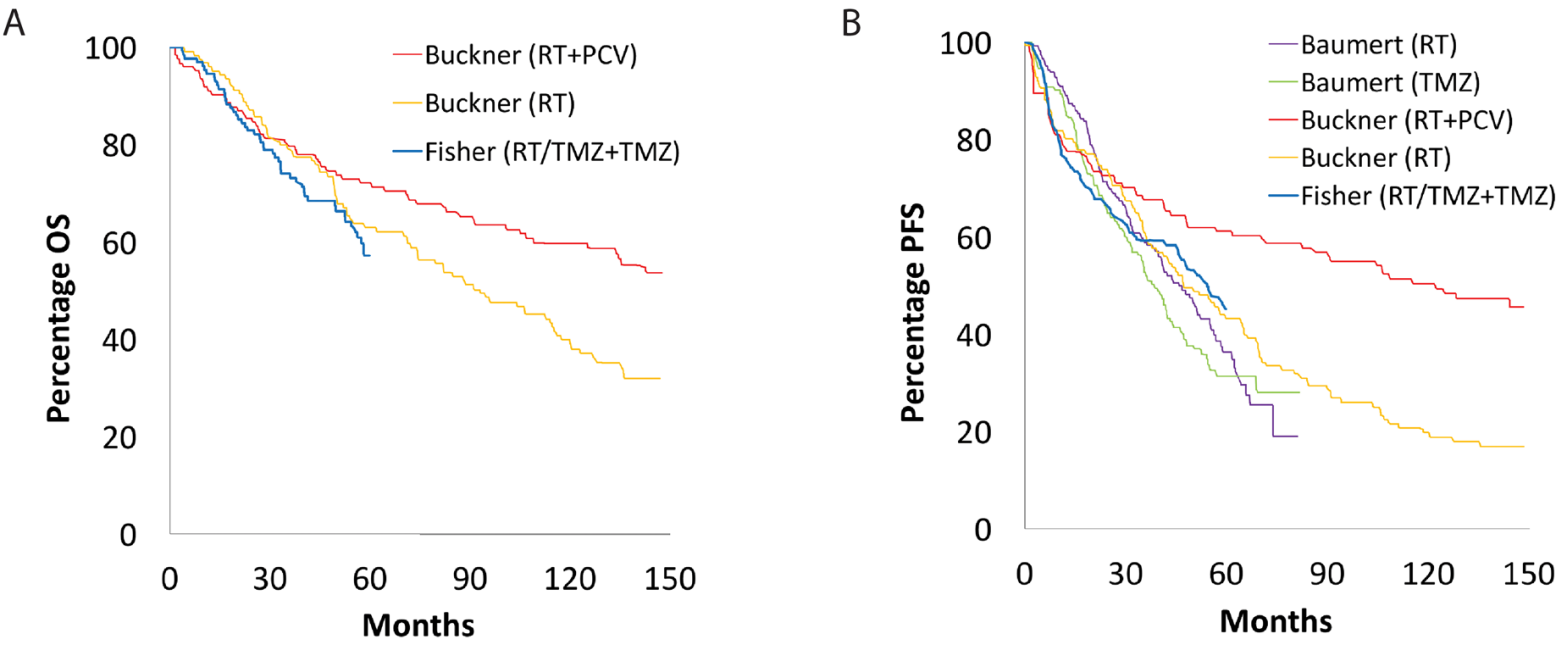
Overall and progression-free survival in patients with low-grade glioma Kaplan-Meier curve showing superimposed studies that assessed **(A)** overall and **(B)** progression-free survival of patients with low-grade glioma.

**Figure 2:**
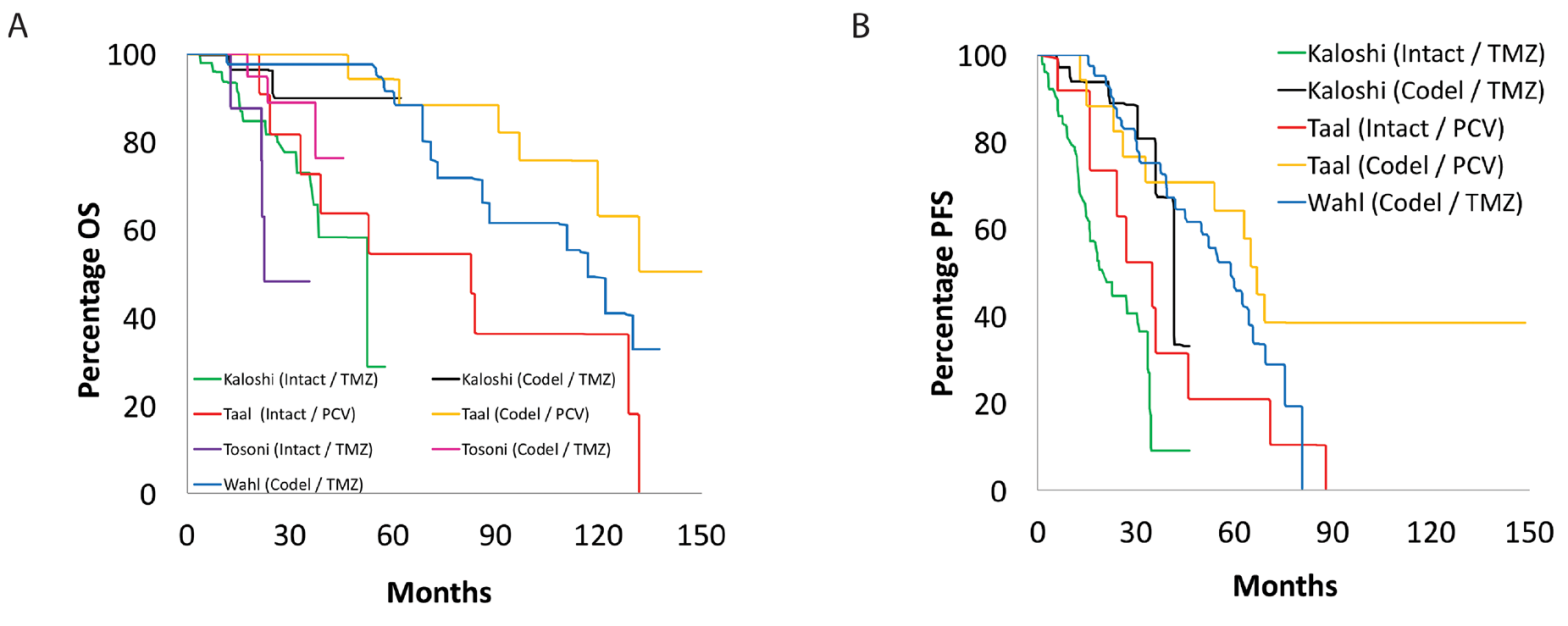
Overall and progression-free survival based on *1p/19q* status Kaplan-Meier curve showing superimposed studies that assessed **(A)** overall and **(B)** progression-free survival of their patients with low-grade glioma based on *1p/19q* status. “Intact” and “codel” refer to intact and co-deleted chromosome 1p and 19q status.

**Figure 3:**
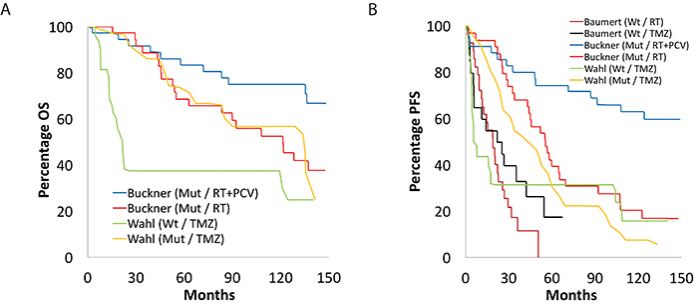
Overall and progression-free survival based on *IDH1* status Kaplan-Meier curve showing superimposed studies that assessed **(A)** overall and **(B)** progression-free survival of their patients with low-grade glioma based on *IDH1* status. “Wt” and “Mut” refer to wild-type and mutated *IDH1*.

Our search of the PubMed database yielded a total of 1,209 papers, of which 19 studies met the inclusion and exclusion criteria: two randomized control trials, thirteen cohort studies, and five retrospective studies (Supplementary Figure 1) [7]. From these studies, 1,720 adult patients were accrued for the systematic review. Table 1 and Table 2 summarize the key findings and survival data for the included studies utilizing PCV and TMZ, respectively. Figure 1, Figure 2, and Figure 3 display superimposed Kaplan-Meier curves from included studies relating OS and PFS to treatment and molecular subtype. Of these 19 papers, ten examined PCV or TMZ as a salvage agent at progression following RT, and were excluded from analysis.

**Table 1:** Treatment of low-grade glioma using PCV

Study	n	Molecular Subtype	Treatment	Median OS (months)	Median PFS (months)	Median OS (5-year %)	Median PFS (5-year %)
Buckner et al., 2003 [8]	28						
		All	PCV with RT			89.0	
Buckner et al., 2016 [9]	251						
		All All *IDH1* Wt *IDH1* Mt	RT PCV with RT PCV with RT PCV with RT	94.0 61.2 157.2	48.0 124.8	63.0 72.0	44.0 61.0
Lebrun et al., 2007 [10]	33						
			PCV			75.0	
Stege et al., 2005 [11]	21						
		All	PCV		24.0 >		
Taal et al., 2015 [12]	32						
		All *1p/19q* Intact *1p/19q* Codeleted	PCV PCV PCV	120.0 83.0 NR	46.0 35.0 67.0		

Abbreviations: OS – Overall Survival, PFS – Progression-Free Survival, RT – Radiation Therapy, PCV – procarbazine, CCNU (lomustine), and vincristine, Mt – Mutant, Wt- Wildtype.

**Table 2:** Treatment of low-grade glioma using TMZ

Study	n	Molecular Subtype/Histology	Treatment	Median OS (months)	Median PFS (months)	Median OS (5-year %)	Median PFS (5-year %)
Baumert et al., 2016 [13]	477						
		All All	RT TMZ		46.0 39.0		40.2 28.9
Dubbink et al., 2009 [14]	49						
		All *1p/19q* Intact *1p/19q* Codeleted	TMZ^*^ TMZ^*^ TMZ^*^	11.0 48.0 98.0		89.0	
Fisher et al., 2015 [15]	129						
		All	TMZ with RT		54.0	57.1	
Hoang-Xuan et al., 2004 [16]	59						
		All	TMZ				
Kaloshi et al., 2007 [17]	149						
		All	TMZ		28.0		
Kesari et al., 2009 [18]	44						
		All *1p/19q* Intact *1p/19q* Codeleted	TMZ TMZ TMZ	> 72.0	38.0 34.0 45.0	73.0	34.0
Koekkoek et al., 2016 [19]	53						
		All	TMZ	39.1		20.0	
Kouwenhoven et al., 2006 [20]	54						
		All	TMZ^*^	81.0			
Levin et al., 2006 [21]	28						
		All	TMZ		31.0		
Pace et al., 2003 [22]	43						
		All	TMZ^*^		10.0		
Quinn et al., 2003 [23]	41						
		All OD OA	TMZ^*^ TMZ^*^ TMZ^*^		22.0 22.0 14.1		
Taal et al., 2011 [24]	58						
		All *1p/19q* Intact *1p/19q* Codeleted *IDH1* Wt *IDH1* Mt	TMZ^*^ TMZ^*^ TMZ^*^ TMZ^*^ TMZ^*^	14.0 12.0 17.0 16.0 12.0	8.0	NR	22.0
Tosoni et al., 2008 [25]	30						
		All	TMZ		21.8		
Wahl et al., 2017 [26]	120						
		All A OD OA *1p/19q* Codeleted *IDH1* Wt *IDH1* Mt	TMZ TMZ TMZ TMZ TMZ TMZ TMZ	116.4 85.2 129.6 68.4 116.4 21.6 134.4	45.6 39.6 55.2 32.4 58.8 7.2 43.2		

Abbreviations: OS – Overall Survival, PFS – Progression-Free Survival, A - Astrocytoma, OA – Oligoastrocytoma, OD – Oligodendroglioma, RT – Radiation Therapy, TMZ – Temozolomide, Mt – Mutant, Wt- Wildtype.

^*^TMZ administered after progression with either prior chemotherapy or radiation therapy.

### Evidence for use of PCV in patients with LGG

We identified five studies in which survival outcomes for patients with LGG treated with PCV were reported (Table 1) [8–12]. These studies included 365 patients with a median age of 43.5 years (age range: 36.0-46.5). Of these, four reported survival data meeting our designated requirements for analysis, resulting in a cohort of 344 patients for study with a median age of 43.75 years (age range 36.0-46.5) [8–10, 12]. Taal et al. (2015) reported a median OS of 120 months when using PCV alone [12]. Buckner et al. (2016) reported a median PFS and OS of 48 and 94 months when RT was administered alone, respectively [9]. The median PFS for PCV alone was 32.5 months (PFS range: 19.0-46.0) [10, 12]. PFS when administering PCV in combination with RT was 124.8 median months [9]. Only one study reported 5-year PFS within this context, with 44% and 61% PFS for RT and a combination of RT and PCV, respectively [9].

### Evidence for use of PCV in patients with histologically subtyped LGG

None of the trials identified in our study reported histologic subtype and OS or PFS following treatment with PCV.

### Evidence for use of PCV in patients with molecular-subtyped LGG

Taal and colleagues reported a median PFS and OS of 35 months and 83 months, respectively, for patients with intact 1p/19q [12]. Median PFS for patients with 1p/19q codeletion was 67 months; median OS was not reached for patients in this cohort.

Buckner and colleagues treated patients with newly diagnosed high-risk LGG with PCV after radiation therapy at the time of initial diagnosis [9]. *IDH1* R132H mutations were detected in 35 of 57 patients (61%) in the group that received radiation therapy alone and in 36 of 56 (64%) in the group that received radiation therapy plus chemotherapy. In this cohort, 78% of patients were diagnosed with oligodendroglioma, 54% with oligoastrocytoma, and 48% with astrocytoma. OS was reported as 61.2 and 157.2 median months for patients with wild-type and mutant IDH1, respectively.

### Adverse events for patients with LGG treated with PCV

Completion of therapy was an issue in the study of Taal et al., in which only sixteen patients (50%) completed six cycles [12]. Toxicities encountered in all studies included bone marrow toxicity, diarrhea, fatigue, and hepatotoxicity. Grade III and IV toxicities were encountered in 47% and 3% of patients, respectively. In the study of Lebrun and colleagues, 9.1% of 33 patients developed a grade III or IV hematological toxicity [10]. Buckner and colleagues (2003) reported grade III/IV leukopenia and thrombocytopenia in 75% and 64% of patients, respectively [8]. Buckner reported grade III and grade IV blood or bone marrow toxicity in 41.6% and 9.6% of 125 patients, respectively [8]. In contrast, patients in the radiation-alone treatment group had 0.8% and 0% grade III and IV blood or bone marrow toxicity.

### Evidence for use of TMZ with LGG

Fourteen studies assessing survival outcomes with TMZ met our criteria for inclusion (Table 2) [13–26]. These studies included 1,355 patients with a median age of 41 years (age range: 38-49). Of these, six studies reported survival data meeting our designated requirements for analysis, which resulting in a cohort of 933 patients for study with a median age of 41.5 years (age range 38-49) [13, 15, 17, 21, 25, 26]. A median OS of 116.4 months was found when administering TMZ on its own [26]. A median 5-year OS of 57.1% was reported when combining TMZ and RT [15].

An open-label, phase 3 European and Canadian intergroup study randomized adult patients with LGG and at least one high-risk feature to either conformal radiotherapy (up to 50.4 Gy) or dose-dense oral TMZ (for a maximum of 12 cycles). The median PFS for RT alone was 46 months, while median PFS for TMZ alone was 31.0 months (PFS range: 21.8-45.6) [13, 17, 21, 25, 26]. One study reported median PFS of 54 months when administering TMZ in combination with RT [15]. Five-year PFS was found to be 40.18% when using RT alone [13]. Median 5-year PFS for TMZ alone was 28.92% [13].

### Evidence for use of TMZ in patients with histologically subtyped LGG

Only one study reporting on the use of TMZ in patients with LGG included data on histological subtype. Wahl *et al* reported an OS of 129.6, 85.2, and 68.4 median months for oligodendrogliomas, astrocytomas, and oligoastrocytomas, respectively [26]. The median PFS was 55.2, 39.6, and 32.4 months, for oligodendrogliomas, astrocytomas, and oligoastrocytomas, respectively [26].

### Evidence for use of TMZ in patients with molecular-subtyped LGG

Only one study reporting on the use of TMZ in patients with LGG included data on molecular subtype. A median PFS and OS of 58.8 and 116.4 months were reported for 1p/19q codeleted patients, respectively [26]. In terms of IDH1, median PFS and OS were 134.4 and 43.2 months for patients with mutant IDH1, respectively, and 21.6 and 7.2 months for patients with wild-type IDH1, respectively [26].

### Adverse events for patients with LGG treated with TMZ

TMZ chemotherapy was also accompanied by adverse effects in all six studies included for analysis. Of 234 patients, Baumert and colleagues reported that 34 (15%) had at least one dose reduction, which was due to hematological toxicity in 19 patients (8%), non-hematological toxicity in nine patients (4%), and for other reasons in 11 (5%) patients. Overall, grade 3–4 haematological toxicity was recorded in 22 (9%) of 235 patients in the safety population who received temozolomide, compared with one (<1%) of 228 patients in the safety population in the radiotherapy group. Kaloshi and colleagues reported that patients treated with TMZ chemotherapy tolerated it well, with 7% grade III and 8% grade IV myelosuppression. Toxicities encountered in all studies included myelosuppression (including leukopenia, neutropenia and thrombocytopenia) and gastrointestinal complaints (mild nausea, emesis, and constipation).

## DISCUSSION

Treatment of patients with LGG is directed toward improving the time to malignant transformation and overall survival, while limiting treatment-associated morbidities and neurologic disability. Objectives for surgery in this patient population include obtaining tissue for diagnosis, improving the quality of life through relief of focal deficits or improved seizure control, and cytoreduction. In a prospective study of patients with LGG with surgeon-determined gross total resection, the presence of residual disease on post-operative imaging, astrocytic histology, and preoperative tumor size were prognostic factors for PFS [13]. In a retrospective population-based parallel cohort study, early surgery afforded a survival benefit compared to biopsy and watchful waiting in patients with low-grade glioma [27].

The utility of involved field radiation therapy is patients with LGG has been clarified by three independent prospective trials. In an intergroup phase III prospective randomized clinical trial of low- versus high-dose radiation therapy in adults with LGG conducted by the North Central Cancer Treatment Group (NCCTG), Eastern Cooperative Oncology Group (ECOG) and the Radiation Therapy Oncology Group (RTOG), no survival difference was found between patients treated with 54 Gy and 65 Gy, with lower doses tending to be associated with less treatment-related toxicities [28]. Similarly, the EORTC found no difference in survival in patients treated with 45 Gy or 59.4 Gy, again with less adverse treatment-related affects in the former group [29]. The EORTC 22845 randomised trial found that early versus delayed RT confered a significant advantage in PFS, but confered no benefit in OS [30]. These findings led many clinicians to reserve radiotherapy for disease progression.

Previous studies from NCIC, the Temodal Brain Tumor Group, and EORTC, have shown a benefit to patients harboring a recurrent anaplastic oligodendroglioma and astrocytoma with both PCV and TMZ [24, 31–34]. The benefit of chemotherapy in this patient group was found to be more frequent and durable responses in patients with histologically classified oligodendroglioma, particularly in those with combined 1p/19q loss, compared with those with an astrocytoma. Four trials investigated adjuvant chemotherapy in addition to radiotherapy. Three of these trials investigated PCV; histologic criteria were used to determine eligibility, with enrollment of patients with anaplastic oligodendroglioma in two of the trials [35, 36], and enrollment of patients with low-grade glioma in the other [9]. The fourth trial investigated concurrent and adjuvant TMZ in patients with anaplastic glioma with intact chromosomes 1p and 19q [37, 38]. All four trials found improvements in outcome with the addition of chemotherapy to radiotherapy [9, 35, 36, 38]. Both the NCIC and EORTC trials found the addition of PCV chemotherapy to RT in patients with anaplastic 1p and 19q co-deleted tumors to result in improved benefit [39, 40]. These trials identified three candidate predictive markers for benefit from adjuvant PCV: IDH mutations, CpG island methylated phenotype, and MGMT promoter methylation [31, 32].

Monotherapy with PCV or TMZ chemotherapy or RT has been investigated by RTOG 0424 (PCV or TMZ versus RT for anaplastic glioma [15]) and EORTC 22033-26033 and NOA-8 (TMZ versus RT in LGG with at least one high-risk feature [13, 41]). Both trials failed to show improvement in outcome after initial treatment with chemotherapy alone, with the suggestion as well of decreased survival after initial chemotherapy in some patient subgroups). In the absence of the results of a trial that formally compares chemotherapy alone to combined chemotherapy and RT, it is likely most reasonable to conclude that combination therapy improves survival compared with single modality treatment.

Recent molecular studies have shown that IDH wild-type low-grade glioma more closely approximates an early stage of primary glioblastoma than an IDH-mutant LGG, and harbours a particularly unfavourable natural history [4]. These findings could be considered as biological support for the recommendation that these patients be treated with adjuvant temozolomide, as has been articulated by the CATNON authors. Conversely, the data predict that patients harbouring an IDH mutant LGG with codeletion of chromosomes 1p and 19q are likely to derive the greatest benefit from adjuvant therapy with PCV. These data offer compelling reason to recommend treatment with radiation followed by adjuvant PCV in patients with an IDH mutant, 1p19q-codeleted tumour. That being said, there is not enough evidence to say RT and TMZ are an inferior option, as this is primarily due to lack of trials investigating this subject matter.

There is the further concern that TMZ chemotherapy may direct these tumors toward a hypermutator phenotype at the time of progression [42]. It is unclear if the same risk exists with PCV therapy. More critically, these findings were identified through study of a small cohort of patients, and need to be validated in a larger treatment group.

As patients with LGG often experience long periods of disease stability, the effect of therapy on health-related quality of life (HRQoL) is all the more critical. While the relatively lesser toxicity of TMZ compared to PCV has led many practitioners to prefer TMZ for their patients with low-grade glioma [43], a recent systematic review of patient-reported HRQoL bemoaned the paucity and heterogeneity of reporting of HRQoL in the LGG literature [44]. A longitudinal study of HRQoL in patients with LGG based on patient self-reporting found that patients with LGG had worse physical role functioning and general health perceptions at long-term follow-up (on average, twelve years following diagnosis) than healthy matched controls, independent of treatment type [45]. No significant differences in HRQoL or global cognitive functioning were seen in patients with high-risk LGG randomized to treatment with TMZ or radiation in EORTC 22033-26033 [46]. While Pace and colleagues performed QoL testing on patients receiving salvage chemotherapy with PCV or TMZ for radiographic progression of LGG, their analysis did not allow for comparison of results from patients in these two subgroups [22]. We were unable to find any other studies in the literature reporting QoL measures on patients with LGG who were treated with PCV.

It is valuable to recollect that the only Level 1 evidence for TMZ in LGG offered by the literature demonstrates the inferiority of TMZ monotherapy to radiation therapy in this patient cohort. Our understanding of the role of TMZ compared to PCV in the treatment of patients with lower-grade glioma will be informed by the CODEL (ALLIANCE-N0577-CODEL) trial, which has reopened as a two-arm comparison of radiation therapy with adjuvant PCV vs. radiation therapy with concurrent and adjuvant TMZ in patients with 1p/19q-codeleted anaplastic (Grade III) oligodendroglioma; whether these findings will be generalizable to patients with Grade II codeleted tumors is unclear. Similarly, in interim results from the CATNON trial (EORTC study 26053-22054), in which patients with newly diagnosed non-co-deleted anaplastic glioma were randomized to RT alone, RT with adjuvant TMZ, or RT with concurrent TMZ with or without adjuvant TMZ, adjuvant temozolomide chemotherapy was found to be associated with a significant survival benefit compared to RT alone. The data was not yet mature at the time of planned interim analysis to determine superiority between concurrent and adjuvant TMZ versus adjuvant TMZ alone. Unfortunately, IDH status was not reported with the interim analysis for the patient cohort enrolled in CATNON, but will be included in the final analysis. Their findings will likely represent results from a mixed population of IDH wild-type and mutated patient; in fact, the quick separation of the survival curves for patients treated with RT alone versus RT with adjuvant TMZ (within one year of treatment), and the lower than expected number of tumors with MGMT promoter methylation (a finding typical for IDH-mutant LGG), suggest that many of these patients will be found to harbour IDH wild-type tumors.

Our study does not allow us to answer a number of critical real-time problems. For example, how should we advise the patient who has undergone gross total resection of an LGG that harbours histologic, molecular, or demographic features of a high-risk lesion? Some authors have advised that patients who have undergone GTR of an LGG (i.e. no residual FLAIR signal abnormality) may be followed expectantly (a “wait and see approach”) and treated if found to have radiographic recurrence [47]. It is unlikely that future trials will include a subgroup that is randomized to treatment with surgical resection alone to allow us the answer to this question. Similarly, the literature reporting on the use of adjuvant therapy in patients with LGG lacks granularity. Should PCV or TMZ be recommended as salvage therapy for a patient who has tumour recurrence following distant radiotherapy for a high-risk LGG without its histological progression? Should PCV or TMZ be used in a patient found to have an intermediate risk lower-grade glioma, for example, a Grade III IDH-mutated, 1p and 19q intact tumour? What patient presentation does a combination of RT and TMZ prove to be the more valuable option of treatment?

Finally, it it is worthwhile to note some of the critical limitations of the data included in our review. For example, these studies all predate the recognition of the intermediate risk LGG represented by tumors harboring an IDH mutation, but lacking 1p and 19q codeletion, and harboring instead deletion of ATRX. In Figure 2, patients captured within the 1p19q-intact cohort may include both this intermediate subgroup and higher-risk patients with IDH wild-type tumors. Conversely, in Figure 3, patients included within the IDH mutant subgroup may include patients with both lower-risk IDH mutant, 1p and 19q co-deleted tumours, as well as patients with intermediate IDH mutant, 1p and 19 intact, ATRX-deleted tumors.

For now, the data suggest that for patients harboring a tumor with an unfavorable natural history, such as those with intact 1p/19q and wild-type IDH1, TMZ and RT may be the best option. Conversely, the data suggest that patients with biologically favorable LGG are likely to derive the most significant benefit from RT and adjuvant PCV. A prospective trial directly comparing PCV and TMZ in patients with high-risk low-grade glioma is needed.

## MATERIALS AND METHODS

This systematic review was conducted according to PRISMA guidelines (Supplementary Figure 2) [7]. The primary literature search was conducted via PubMed for articles published between January 1, 1995 and May 1, 2017, using the search terms, “Low-grade glioma AND temozolomide OR TMZ”, “Low-grade glioma AND PCV OR procarbazine/lomustine/vincristine”, and “Low-grade glioma AND chemotherapy”. References from relevant articles were searched. Level I, II, and III studies were included if published in English, consisted of patients 16 years of age and older, noted LGG subtype, progression-free survival (PFS) and overall survival (OS) as primary or secondary end points, and treatment course. Exclusion criteria included pediatric patient populations and lack of differentiation of survival data based on treatment or glioma-subtype. Data including study design, patient population demographics, histology, molecular subtype, PFS, OS, and treatment regimens and durations were extracted. Levels of evidence were categorized as follows: level I (properly powered and conducted randomized-control trial), level II (well-designed controlled trial without randomization; prospective comparative cohort trial), and level III (retrospective cohort study) [48]. Figures including superimposed Kaplan-Meier curves were generated using Digitizelt (Bormisoft, Braunschweig, Germany, http://www.digitizeit.de), a software designed to digitize scanned graphs and charts into (x,y)-data. Software errors in data recognition were manually edited by the authors (K.H. and S.D.).

## CONCLUSIONS

In patients harboring a tumor with an unfavorable natural history, such as those with intact 1p/19q and wild-type IDH1, RT/TMZ plus adjuvant TMZ may be the best option. Conversely, patients with biologically favorable high-risk LGG are likely to derive the most benefit from RT and adjuvant PCV. While unlikely due to the resources and time required, a prospective trial directly comparing PCV and TMZ in patients with high-risk low-grade glioma is needed.

## SUPPLEMENTARY MATERIALS FIGURES AND TABLES




