# Supplementary material for: Procarbazine, CCNU and vincristine (PCV) versus temozolomide chemotherapy for patients with low-grade glioma: a systematic review
Source: Oncotarget. 2018 Sep 14;9(72):33623–33. doi: 10.18632/oncotarget.25890 (PMC6154749; doi:10.18632/oncotarget.25890)
Supplement: Supplementary file 1 [file oncotarget-09-33623-s001.pdf]

Procarbazine, CCNU and vincristine (PCV) versus temozolomide chemotherapy for patients with low-grade glioma: a systematic review

SUPPLEMENTARY MATERIALS

PRISMA Flow Diagram

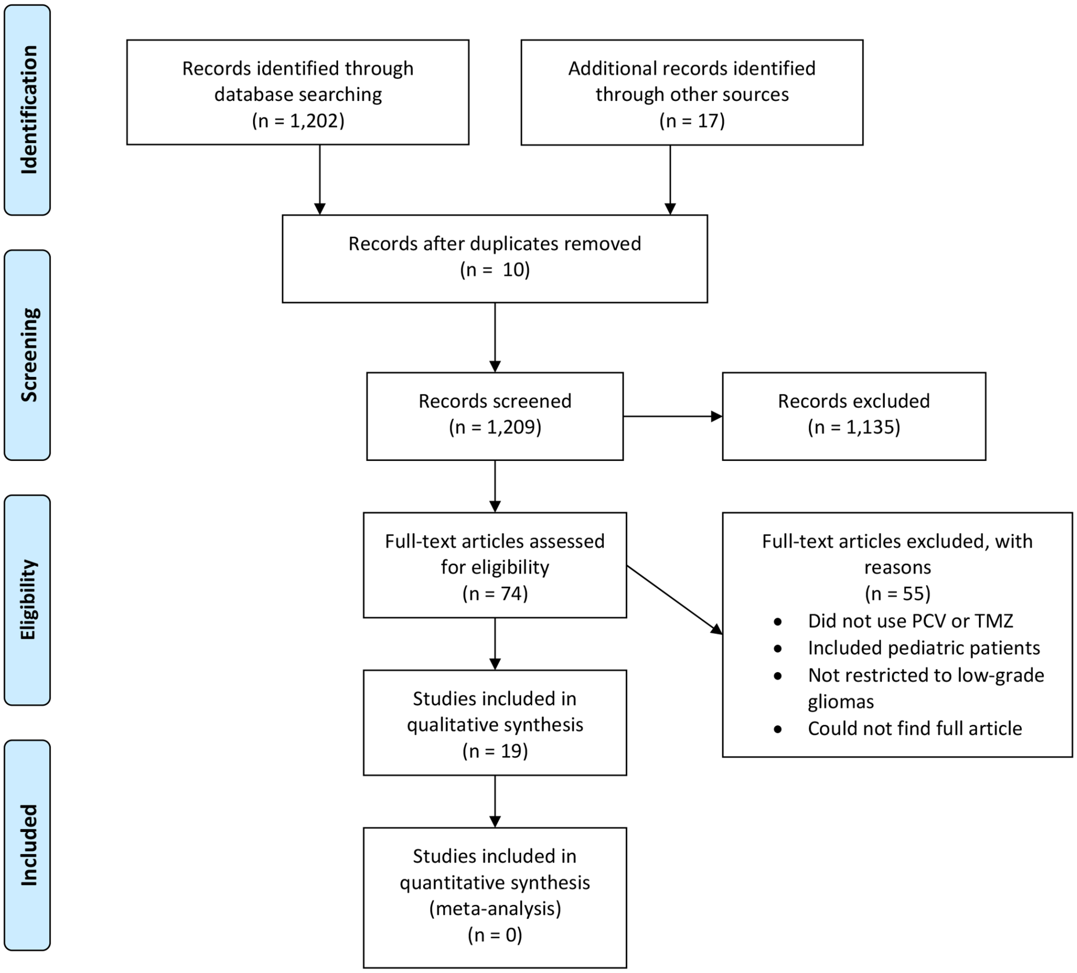

From: Moher D, Liberati A, Tetzlaff J, Altman DG, The PRISMA Group (2009). *P*referred *R*eporting *I*tems for *S*ystematic Reviews and *M*eta-*A*nalyses: The PRISMA Statement. PLoS Med 6(6): e1000097. doi:10.1371/journal.pmed1000097

For more information, visit www.prisma-statement.org.

Supplementary Figure 1: PRISMA flow diagram of literature review process. Flow diagram detailing the literature review process, including initial screening, reasons for excluded papers, and final papers meeting inclusion criteria.

Supplementary Figure 2: PRISMA checklist. Checklist describing sections of manuscript that abide by PRISMA requirements.

See Supplementary Figure 2
